# Public Engagement in Digital Recommendations for Promoting Healthy Parental Behaviours from Preconception through the First 1000 Days

**DOI:** 10.3390/ijerph20021329

**Published:** 2023-01-11

**Authors:** Giulia Cinelli, Ileana Croci, Francesco Gesualdo, Elisabetta Pandolfi, Kiersten Pilar Miller, Alberto Eugenio Tozzi

**Affiliations:** Predictive and Preventive Medicine Research Unit, Bambino Gesù Children’s Hospital, IRCCS, 00165 Rome, Italy

**Keywords:** 1000 days, digital communication, innovation

## Abstract

Web-based digital interventions may play a central role for health promoting strategies in the first “1000 days”, from conception through the first 2 years of life. We developed a web platform providing evidence-based recommendations in the first 1000 days through short videos, and we studied engagement by users from preconception through parenthood in the second year of life. We described the access to videos by topic and used a multilevel model to explore the user characteristics associated with access to the video recommendations. Overall, breastfeeding, physical activity and nutrition were the most popular topics (normalized views: 89.2%, 87.2% and 86.4% respectively), while content on paternal health and smoking and alcohol was less engaging (37.3% and 42.0%). Nutrition content was the most viewed in the preconception period and during the first two trimesters of pregnancy. Nutrition and breastfeeding were also the most popular topics for users with children less than 2 years old. Higher levels of health literacy were associated only with child health content. The study findings indicate that digital strategies should be adapted according to the time period in the first 1000 days. Alternative digital promotion strategies for the less engaging topics should be considered.

## 1. Introduction

The “first 1000 days” refers to a crucial period of a child’s life, starting from conception through the first 2 years of life [1,2]. Research has shown that during this period, a variety of health-promoting actions, including interventions aimed at improving behaviours, and optimisation of therapy for parents’ underlying conditions, can establish the foundations for optimal health, growth and neurodevelopment across the lifespan of their offspring [3].

The existing evidence regarding the efficacy of health interventions in the first 1000 days includes actions addressing several health areas (alcohol, smoking, nutrition, physical activity, folic acid supplementation), targeting multiple subjects (parents and/or child) and taking place during different time periods (preconception, children under 2 years of age) [4,5,6,7]. Therefore, the systematic implementation of these interventions requires multiple complementary strategies to efficiently reach the right targets at the right time.

Health promoting strategies in the first 1000 days are usually implemented through in-person recommendations during medical encounters and information campaigns for the general public [8]. Ideally, in person counselling should cover a wide range of topics with many details that would require multiple encounters of reasonable length. An optimal implementation of these recommendations, however, is far from being achieved, mainly due to time constraints, leaving enormous room for improvement and a potential for positively affecting population health.

As of January 2022, in Italy, there were about 50 million Internet users, representing over 84% of the total population [9]. The percentage of people in Italy using the web to seek medical and health information has constantly increased in the past few years [10] and the Internet was detected as the most common source for this purpose also during the COVID-19 crisis [11]. As highlighted by the COVID-19 pandemic [12], web-based digital interventions may play an important role in providing information to the general public regarding the first 1000 days, which complement the existing strategies to support families in adopting healthy behaviours and making appropriate decisions for their health [13,14,15]. In addition, digital interventions may be tailored to target specific profiles, thus reducing the background noise of information overload.

Nearly 90% of parents globally use the web to learn about their pregnancy or child’s health [16,17,18,19]. The most common searches by mothers during pregnancy are regarding foetal development, nutrition and healthy lifestyles, pregnancy complications and physical symptoms (nausea, pain and bleeding), stages and physiology of childbirth, child sleep and breast or formula feeding [13,16,17,19,20,21,22]. Right after birth, women tend to search for online information on child health [22,23], mostly focusing on paediatric conditions, diagnosis or treatment, parenting advice [24] or general health topics, such as paediatric nutrition or development [23].

Unfortunately, it is known that online health content is not always valid or evidence-based [25,26]. Since the information found on the web may be poor and inaccurate, it is likely that it may not have a positive impact on parents’ behaviour [25]. For this reason, strategies to improve the quality of online information related to preconception, pregnancy and child health should be considered [25].

In 2019, the Italian Ministry of Health issued a document which included a collection of recommendations based on the existing evidence regarding health promoting actions in the first 1000 days [27]. Based on this document, we developed a web platform which hosted short videos on topics relevant to health promoting actions with the aim of providing digital, evidence-based recommendations during the first 1000 days to the general public. We collected information on the engagement with different topics and analysed visitor profiles. 

The objective of this study is to describe the characteristics and the interactions of users engaged in a web platform providing evidence-based video recommendations on health topics from preconception through the first 1000 days. In addition, we aim to investigate the determinants of engagement with specific topics, including health literacy (HL) level and sociodemographic information.

## 2. Materials and Methods

### 2.1. Setting

The Bambino Gesù Children’s Hospital is a large paediatric research hospital located in Rome, Italy. The hospital is engaged in several digital information projects on health promotion for child health. Digital multimedia content is offered on the web page of the hospital and on social media. Digital content is offered in Italian only and the reach is approximately 3 million people per week. Web visitors and social media followers include people from across the entire country.

### 2.2. Platform Development

A brand-new web platform for this project was developed by a multidisciplinary group including paediatricians, epidemiologists, nutritionists, IT engineers and digital communication experts. Development of the content was based on a comprehensive review of existing evidence issued by the Italian Ministry of Health [27]. We extracted 18 recommendations from those targeting the general public and invited scientific experts in those fields to give a short talk that was video recorded with professional standards. Evidence-based recommendations were explained in plain language and with a positive communication style.

The list of the recommendations, the corresponding videos and the target audience are illustrated in Table 1. A total of 44 videos were uploaded to the platform (examples of the videos are shown in Table S1). As the number of videos was not similar across the different topics, the number of views for each topic was normalized dividing the number of views by the number of videos.

Access to the web platform was granted by a free subscription requiring registration with an email address and an acknowledgement of informed consent (Figure S1). The platform was promoted through social networks of the Bambino Gesù Children’s Hospital and other local maternal health communities.

### 2.3. Subjects and Study Design

Prior to the registration, all participants signed an electronic informed consent regarding data protection and data treatment. After this first step, participants confirmed their registration by email and filled in a questionnaire including sociodemographic information as well as a specific section which evaluated their HL level. We also recorded whether the user was planning a pregnancy, the gestational age if experiencing a pregnancy, or whether they had a child younger than 24 months. Users neither planning a pregnancy, nor experiencing a pregnancy nor having children in the first 2 years of life were also included. After completing the questionnaire, users were allowed to browse the website and the video content. Searches for specific information were facilitated by a search query system on the home page which allowed users to seek video according to relevant time period categories (preconception, pregnancy, post-partum) and topic.

We monitored the web analytics for each video in the period from October 2019 to June 2022 to evaluate how frequently each video was sought out.

### 2.4. Health Literacy Evaluation

HL is a complex concept involving the ability to use and interpret texts, documents, and numbers related to the healthcare environment. The questionnaire used in our study consisted of a combination of two validated questionnaires [28,29]. An additional question on “The ease of using information given by the physician to make decisions about the pathology” was added. All the answers were based on a Likert scale.

Once the study was closed, we carried out an exploratory factor analysis using a polychoric correlation matrix to take into account the ordinal nature of the score [30]. As the six variables were loaded on one single factor with moderate factor loadings (range 0.33–0.65), the unweighted individual scores were added to obtain the total HL score. The acceptable ordinal reliability alpha (0.60) confirmed the internal consistency of the scale [30]. HL theoretical score ranged from 6 to 27, with higher values indicating a greater ability to interpret and understand health information. We categorised the score in three different levels (low, medium, high) based on the tertile distribution and this variable was used in the analysis.

### 2.5. Statistical Analysis

We performed a descriptive analysis of the sociodemographic characteristics of users and of their HL level. To describe the level of engagement in specific topics, we grouped the videos into 7 categories as illustrated in Table 1. We then categorised users into 7 groups according to the time period they belonged: preconception, first, second and third trimester of pregnancy; parent of child less than 12 months of age or less than 24 months of age; and an additional category for users not planning a pregnancy, not experiencing a pregnancy, and not having children in the first 2 years of life, as a reference category. In case a user fell into more than one category, the record was duplicated in order to include the subject in both categories. 

We developed a heatmap illustrating the absolute number of viewings for each video in a certain topic by user category. 

We also developed a multilevel logistic regression model to identify the user profile characteristics associated with the access to at least one of the videos for each topic (outcome). The model included the following variables as predictors: age (<31 years, 31–35 years, 36–40 years, >40 years), regional distribution (northern Italy, central Italy, southern Italy and islands), graduate school degree (no, yes), civil status (unmarried, married, living together, divorced), occupation (full-time, part-time, unemployed), health professional (no, yes), time period (no plan/no pregnancy/no child/child >2 years; preconception, first, second and third trimester of pregnancy; first and second year of life) and HL score (low, medium, high). User IDs were included as a random effect in multilevel models. 

Continuous variables were reported as mean and standard deviation or median and interquartile range, as appropriate. Categorical variables were tabulated as frequencies and percentages. 

Data analysis was carried out using STATA 17 (StataCorp., College Station, TX, USA). Graphical analysis was performed using Tableau 2020.3 (Seattle, WA, USA).

## 3. Results

### 3.1. Users

A total of 187,243 people accessed the web page of the platform and 1569 (0.84%) signed the informed consent. Of those, 270 participants were excluded for the following reasons: 9 did not complete the HL questionnaire, 120 did not provide their socio-demographic data, 109 did not confirm their participation by email, and 32 requested to withdraw from the study and delete their data.

The final analysis was therefore performed on data from the 1299 users who fully registered on the platform. The majority were Italian (n = 1261, 97.1%) and female (n = 1240, 95.5%) and their age ranged from 20 to 66 years. Users’ characteristics are shown in Table 2. Out of the total participants, 380 were parents of children <2 years old (28.3%), 257 were parents of children <1 year old (19.2%), 149 were expectant parents in the first trimester (11.1%), 177 were expectant parents in the second trimester (13.2%), 171 were expectant parents in the third trimester (12.7%), 97 users were planning a pregnancy (7.2%) and 111 were neither planning a pregnancy, nor experiencing a pregnancy, nor having children in the first 2 years of life or having no children at all (8.3%), and Table S2 shows the characteristics of this group, which was used as reference category in the analysis.

### 3.2. Health Literacy

Users showed an HL score of 19.74 ± 3.04 (range: 11–27), with a normal distribution. In particular, 462 (35.6%) scored “low”, 469 (36.1%) scored “medium” and 368 (28.3%) scored “high” levels of HL. The percentages of answers to each question are shown in Table S3. Users with high health literacy levels also had a high education level or were health professionals. 

### 3.3. Videos

**Figure 1:**
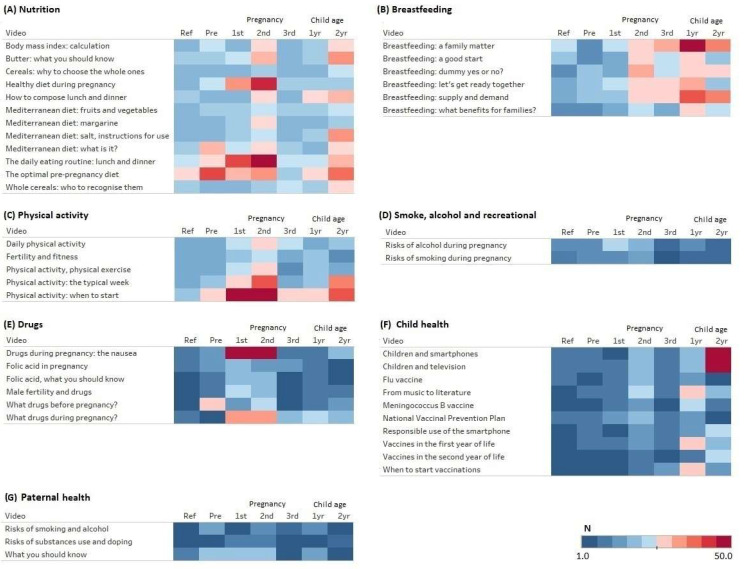
Absolute frequency of video views by topic and users’ category. Ref: not planning a pregnancy, not experiencing a pregnancy, and not having children in the first 2 years of life; Pre: planning for pregnancy; 1st: expectant parents first trimester; 2nd: expectant parents second trimester; 3rd: expectant parents third trimester; 1 year: parents of children <1 year; 2 year: parents of children <2 years.

The videos had 3417 views. Figure 1 shows the frequency of views by user category for the videos included in each of the health topics through a heatmap, and Table 3 shows the normalized number of views adjusted for the number of videos in each topic.

The level of completeness in watching the videos varied from 31% to 66%. The most frequently viewed videos were those regarding nutrition (Figure 1) with a peak for users in the second trimester of pregnancy regarding the general aspects of diet in pregnancy and for users planning a pregnancy regarding diet in the preconception period. A high engagement with nutrition recommendations was also evident for users with children less than 2 years of age. The engagement with breastfeeding recommendations (panel B) started to increase in the second trimester of pregnancy and was highest during the first year of life of the child. Regarding physical activities, users were most engaged with recommendations in the first and second trimesters of pregnancy, with a progressive decline thereafter. Videos including recommendations regarding smoking and drinking alcohol were viewed less frequently than the others. While information regarding pharmaceuticals in pregnancy were mostly accessed in the first and second trimester, information on folic acid was rarely viewed. Topics relevant to child health started to be viewed in the second trimester of pregnancy, the frequency decreased in the third trimester and increased again after birth. Finally, topics relevant to paternal health were rarely viewed. Users not planning a pregnancy, not experiencing a pregnancy or without a child less than 24 months of age rarely accessed any information.

Table 4 shows the results of the multilevel logistic regression model for the variable “period” in which users not planning a pregnancy, not experiencing a pregnancy, and not having children in the first 2 years of life were the reference category. Users in the preconception period and in the first two trimesters of pregnancy showed to be significantly more engaged with videos about nutrition and pharmaceuticals. Additionally, physical-activity-related content was accessed particularly by women in the first and second trimesters. Videos on paternal health, while not often accessed, were most frequently viewed by those in the preconception period. Pregnant women in the third trimester and parents seem to be the least engaged groups in all these topics, and actually they resulted in being significantly less engaged with videos related to smoking, alcohol and recreational drugs than the comparison group. 

Independently from the time period in the first 1000 days, healthcare workers were 3.46 (C.I.: 1.40–8.54) times more engaged with pharmaceutical topics compared to other users. Moreover, users from southern Italy were less engaged with breastfeeding topics (OR: 0.44; C.I.: 0.21–0.95), while physical activity contents was less popular among unemployed users (OR: 0.37; C.I.: 0.15–0.91). Finally, users older than 40 years showed a higher engagement with information on child health than those under 30 (OR: 2.71; C.I.: 1.09–6.70) as well as users with a medium HL score compared to those with a low HL score (OR: 2.05; C.I.: 1.12–3.75). Results are shown in Table S4.

## 4. Discussion

We found that the engagement of users who are offered evidence-based recommendations on health promotion on a digital platform significantly varies over the different time periods of the first 1000 days. We observed that the most engaging topics were breastfeeding, physical activity, and nutrition, while smoking, alcohol and recreational drugs and paternal health were the least popular topics. Compared with users not experiencing a pregnancy or parents of children younger than 2 years, users were most engaged with medication treatments from preconception through the second trimester of pregnancy, as well as nutrition and physical activity in the same time frame. The attention for paternal health was higher than the comparison group in the preconception period. On the other hand, the engagement for content regarding alcohol, smoking and recreational drugs was significantly lower than the comparison group from the third trimester of pregnancy onward. These patterns may be helpful to identify different levels of engagement by topic and adapt communication strategies. 

We also found that digital content on the promotion of health in the first 1000 days provided online is attractive to a public with a high educational level and with a high HL level. Most participants in our study had a university degree and were mostly employed. Moreover, the majority of participants were women of reproductive age, consistent with the mean age of pregnant women expecting their first child in Italy [31]. This result is consistent with other studies indicating that proxy seekers of information on the web tend to be women [32,33], even if it seems that parents’ gender has no influence on whether they searched for health information for their child on the Internet. As previous studies demonstrated that well-educated parents used the Internet to seek out information on their children more than parents with a low-grade education [23], alternative communication strategies should be considered for this population segment, including social networks.

The analysis of engagement of the study participants with digital content offers some appealing insights. We analysed the frequency of access to digital content and the relative engagement of users over the first 1000 days compared with a group not in the first 1000 days. While engaging with recommendations on lifestyles once pregnancy has started is obviously strongly motivated by the responsibility for a new life, health-promoting recommendations in the preconception period are still scarcely considered, although the prevalence of risk factors for adverse pregnancy outcomes in the Italian population is high [34]. Of note, only 7% of users in our study were planning a pregnancy, which is in line with what was previously described regarding poor preconception care by women, probably due to a general lack of valid information sources and awareness of preconception health [8]. 

The number of views of videos on folic acid supplementation, smoking, and alcohol consumption was low and the relative engagement with content on these topics was lower than the comparison group from the third trimester of pregnancy onward. Evidence suggests that folic acid supplementation and avoiding smoking and alcohol in the preconception period have a high impact on foetus and child health [3,35]. Apparently, these topics were not recognized as important by participants in this study. Unfortunately, data from the Italian National Health Institute showed that only one out of five (21.7%) women took folic acid supplementation adequately before getting pregnant [36], as also highlighted by studies directly interviewing Italian women [8,34]. Although we did not collect information on these behaviours in our study, we underline how important it is to reinforce recommendations on these interventions to prevent congenital defects, poor intrauterine growth, and premature delivery [8,34]. 

Another undervalued area for health promotion is paternal health. Male fertility may be affected by some behaviours that can be easily changed with the appropriate recommendations [37] and deserve to be promoted with both parents. The number of male participants in the study was low and it is likely that women may have searched for topics more relevant to their own personal health. However, participants in the study showed a higher engagement with content for this topic in the preconception period compared with the comparison group.

Conversely, a strong engagement with proper diet and physical activity in pregnancy has been observed in several studies investigating women’s informational needs in pregnancy [16,17,20,38] and is confirmed by our results. Of note, the engagement of participants with content on these topics declined in the third trimester of pregnancy and was significantly low in unemployed users compared to workers. These findings confirm what is already described in literature, associating an increased risk of lack of physical activity with unemployment [39].

Breastfeeding was also a popular topic among participants in this study after delivery. Although the WHO recommends exclusive (without any other solid or liquid) breastfeeding for the first 6 months of life, in Italy only 23.6% of infants between 4 and 5 months were exclusively breastfed from birth in 2019, with major differences between northern (44.7%) and southern (16.6%) regions [40]. Since these figures are far from being ideal, and considering are results confirming the scarce engagement of users from southern Italy in breastfeeding recommendations, its promotion remains a priority. The use of digital information resources in this respect may be considered in order to reach out to specific geographical targets.

Finally, child health content referring to vaccinations and screen exposure resulted in being significantly popular between older users with higher HL. In particular, exposure of children less than 2 years of age to smartphones and TV was an engaging topic. Data available on digital technologies in Italy show that about 30% and 60% of parents allow their smartphones to be used by their children younger than 1 or 2 years of age, respectively [41]. This is in strong contrast to the Italian Pediatric Society recommendation on media devices, which strongly recommends against exposure for children under 2 [42], which should be included in the guidelines for child health in the first 1000 days. 

It is possible that topics such as breastfeeding and nutrition were most searched for since little information is provided on these topics by health professionals, while parents may need support since they can represent a day-to-day challenge. Conversely, other topics may be searched for less frequently because users are already familiar with them. Folic acid, alcohol and recreational drugs, for example, are topics that require simple and easy to follow recommendations (supplementation or abstention) and on which awareness campaigns have been largely promoted in the past few years. 

All these findings show how a tool such as the one described in our study not only provides evidence-based recommendations that may be in an accessible format but also offers the possibility of monitoring the engagement by topic and user characteristics. This information may be easily combined with epidemiological findings regarding the prevalence of risk factors in the general population and inform tailored extended strategies through other communication channels.

Our study has some strengths as well as limitations. To our knowledge, this is the first study investigating the engagement with digital content on health promotion recommendations in the first 1000 days, using a specifically designed and evidenced-based platform. We were able to collect data on a large number of users, but since the website required registration and profiling, we may have selected a highly educated population. Additionally, although paternal health is an important pillar of prevention in the preconception period, the engagement with this topic was low possibly due to the small number of males participating in the study. Moreover, the available videos could not cover all of the evidence-based recommendations and that could have influenced the users’ searches. Finally, we did not collect feedback from users, which could have been useful to understand their thoughts on the platform and their specific needs, and we did not follow them up to measure the impact of our intervention.

## 5. Conclusions

In conclusion, the results of our study suggest that digital strategies for promotion of health in the first 1000 days may play a role that should be seriously considered by public health agencies and should be recommended by health providers to their patients. In this context, healthcare professionals should be actively involved in the conception and design of communication tools to guarantee updated evidence-based materials. The findings of our study indicate that digital strategies should also be adapted according to the time period in the first 1000 days and can inform supplemental communication strategies for the highest impact on public health. When developing digital health promoting strategies, it should be taken into account that engagement with some topics may be low and that alternative promotion strategies should be complemented. In this regard, search engine optimization of specialized websites and the use of social networks may help to reach groups of different age, sex and educational level with more finely tailored content. Further studies are needed in order to investigate specific users’ needs and to involve men specifically, which is fundamental in building an effective platform. Additionally, cohort studies are needed to investigate the impact of digital health information on users’ behaviors.

## Figures and Tables

**Table 1:** List of recommendations translated into videos.

Topic	N° of Videos	Recommendation	Preconception Period	Pregnancy	Child
Nutrition	12	Adopting a normocaloric diet	M, F	M	M, F
		Eating 5 servings of fruits and vegetables per day	M, F	M	M, F
		Eating 2–3 servings of seafood per week	M, F	M	M, F
		Maintaining an appropriate BMI	M, F	M	
Breastfeeding	6	Starting breastfeeding as early as possible		M	M
		Maintaining exclusive breastfeeding at least up to 6 months		M	M
		Maintaining breastfeeding after introducing solids			M
Physical activity	5	Engaging in regular physical activity	M, F	M	
		Being physically active throughout the day			M, F
Smoke, alcohol and recreational drugs	2	Avoiding smoking	M, F	M	
		Avoiding drinking alcohol	M, F	M	
Pharmaceuticals	6	Using pharmaceuticals and medications appropriately	M, F	M	
		Taking folic acid supplementation	M	M	
Child health	10	Being appropriately immunized			M, F
		Delaying the introduction of technologies			M, F
Paternal health	3	Male fertility	F		
		Avoiding smoking and alcohol	F		
		Avoiding recreational drugs and doping	F		

F, father; M, mother.

**Table 2:** Users sociodemographic characteristics (n = 1299).

Characteristics	n (%)
Nationality (Italian)	1261 (97.1)
Gender (female)	1240 (95.5)
Age (median, IQR)	35.0 (32.0–39.0)
Age group	
≤30 years	225 (17.3)
31–35 years	468 (36.0)
36–40 years	401 (30.9)
>40 years	205 (15.8)
Region	
Northern Italy	422 (32.5)
Central Italy	594 (45.7)
Southern Italy and Islands	283 (21.8)
Civil status	
Unmarried	168 (12.9)
Married	769 (59.2)
Living together	348 (26.8)
Divorced	14 (1.1)
Educational level	
Secondary school degree or less	26 (2.0)
High school degree	306 (23.6)
Graduate school degree	967 (74.4)
Occupation	
Full-time professional	854 (65.7)
Part-time professional	207 (15.9)
Unemployed	238 (18.3)
Health professional (yes)	308 (23.7)

Values are expressed as number and percentage (n (%)) for categorical variables and median and IQR (M (IQR)) for continuous variables.

**Table 3:** Normalized number of views by topic.

Topic	Number of Videos	Number of Views	Normalized Views
Nutrition	12	1037	86.4
Breastfeeding	6	535	89.2
Physical Activity	5	436	87.2
Smoking, alcohol and recreational drugs	2	84	42.0
Pharmaceuticals	6	378	63.0
Child health	10	837	83.7
Paternal health	3	112	37.3

**Table 4:** Association between users’ time period and video topics.

	Nutrition	Breastfeeding	Physical Activity	Smoke, Alcohol and Recreational Drugs	Pharmaceuticals	Child Health	Paternal Health
Period							
Preconception	**9.4 (2.5–35.8)**	0.6 (0.1–2.3)	3.1 (0.7–14.0)	1.0 (0.4–2.6)	**13.8 (2.0–95.8)**	0.9 (0.2–3.3)	**4.0 (1.6–10.1)**
1st trimester	**4.8 (1.4–15.9)**	0.9 (0.2–3.0)	**8.3 (2.0–35.2)**	1.0 (0.4–2.4)	**24.6 (3.7–63.9)**	0.9 (0.3–2.9)	2.1 (0.9–5.2)
2nd trimester	**7.0 (2.1–23.4)**	2.6 (0.8–8.8)	**9.2 (2.2–38.8)**	0.9 (0.3–2.3)	**9.7 (1.7–57.3)**	2.7 (0.8–8.7)	1.9 (0.7–4.9)
3rd trimester	0.8 (0.3–2.6)	2.3 (0.7–7.5)	0.7 (0.2–2.8)	**0.3 (0.1–0.8)**	1.5 (0.3–7.9)	1.6 (0.5–5.0)	0.9 (0.3–2.5)
1 year of life	0.4 (0.1–1.2)	2.6 (0.8–8.1)	0.4 (0.1–1.6)	**0.3 (0.1–0.9)**	0.4 (0.1–2.1)	2.2 (0.7–6.7)	1.1 (0.4–2.7)
2 years of life	1.1 (0.4–3.2)	0.5 (0.2–1.6)	0.6 (0.2–2.1)	**0.2 (0.1–0.5)**	0.5 (0.1–2.1)	1.4 (0.5–4.0)	0.4 (0.1–1.1)

Multilevel logistic regression model. The model is also adjusted for age, region, educational level, civil status, occupation, health professional, health literacy score. (Ref: No child/child >2 years/No preg/No plan). Values are expressed as odds ratios and 95% confidence intervals (OR (CI 95%)). Statistical significance for *p* < 0.05 (in bold).

## Supplementary Materials

The following supporting information can be downloaded at: https://www.mdpi.com/article/10.3390/ijerph20021329/s1, Table S1: Link to some of the videos (one per topic); Table S2: Characteristics of the reference group: No child/child >2 years/No pregnancy/No planning a pregnancy (n = 111, age range 20–66 years); Table S3: Health Literacy Score’s construction and percentage of answers for each question (n = 1299); Table S4: Association between users’ characteristics and videos’ topic. Figure S1: (a) Website’s landing page; (b) Website’s registration and consent form page; (c) Website’s homepage; (d) Screenshot from one of the video interviews with graphics.
