# Supplementary material for: Public Engagement in Digital Recommendations for Promoting Healthy Parental Behaviours from Preconception through the First 1000 Days
Source: Int J Environ Res Public Health. 2023 Jan 11;20(2):1329. doi: 10.3390/ijerph20021329 (PMC9859030; doi:10.3390/ijerph20021329)
Supplement: Supplementary file 1 [file ijerph-20-01329-s001.zip › ijerph-2027489-supplementary.pdf]

International Journal of
Environmental Research and Public Health

MDPI

Supplementary materials

Table S1. Link to some of the videos (one per topic).

Topic	Title	Link
Nutrition	The optimal pre-pregnancy diet	https://vimeo.com/420993820
Breastfeeding	Breastfeeding: a good start	https://vimeo.com/344058099
Physical Activity	Physical activity: when to start	https://vimeo.com/344042663
Smoke and Alcohol	Risks of alcohol during pregnancy	https://vimeo.com/420995071
Drugs	Folic acid in pregnancy	https://vimeo.com/402472298
Child health	National Vaccinal Prevention Plan: what it is	https://vimeo.com/467712757
Father health	Male fertility: risks of smoking and alcohol	https://vimeo.com/363756711

Table S2. Characteristics of the reference group: No child/child>2y/No pregnancy/No planning a pregnancy (n=111, age range 20-66 years).

Characteristics	n (%)
Nationality (Italian)	106 (95.5)
Gender (female)	102 (91.9)
Age (median, IQR)	39.0 (31.0 - 47.0)
Age group	
≤30 y	26 (23.4)
31-35 y	18 (16.2)
36-40 y	17 (15.3)
41-45 y	19 (17.2)
>45 y	31 (27.9)
Region	
Northern Italy	43 (38.7)
Centre Italy	47 (42.3)
Southern Italy and Islands	21 (18.9)
Civil status	
Unmarried	34 (30.6)
Married	56 (50.5)
Living together	15 (13.5)
Divorced	6 (5.4)
Educational level	
Secondary school degree or less	5 (4.5)
High school degree	21 (18.9)
Graduate school degree	85 (76.6)
Occupation	
Full-time professional	77 (69.4)
Part-time professional	15 (13.5)
Unemployed	19 (17.1)
Health professional (yes)	69 (75.0)

Values are expressed as number and percentage (n (%)) for categorical variables and median and IQR (M (IQR)) for continuous variables.

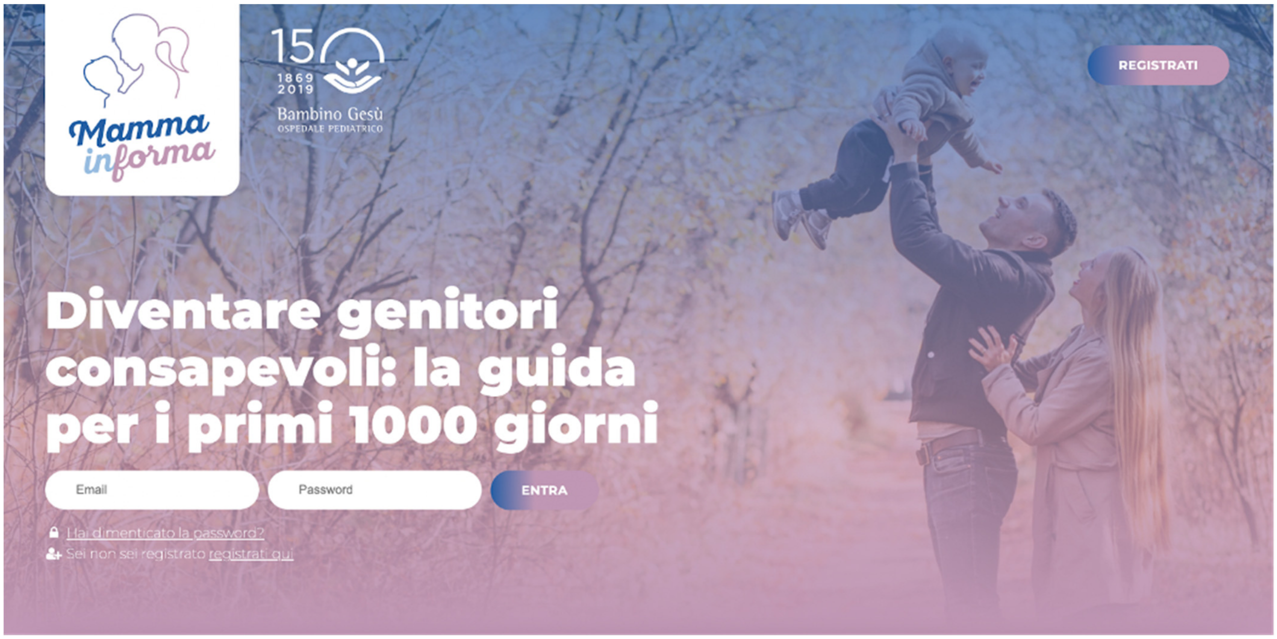
Mamma informa
15
1869
2019
Bambino Gesù
OSPEDALE PEDIATRICO
REGISTRATI
Diventare genitori consapevoli: la guida per i primi 1000 giorni
Email
Password
ENTRA
Hai dimenticato la password?
Sei non sei registrato registrati qui

(a)

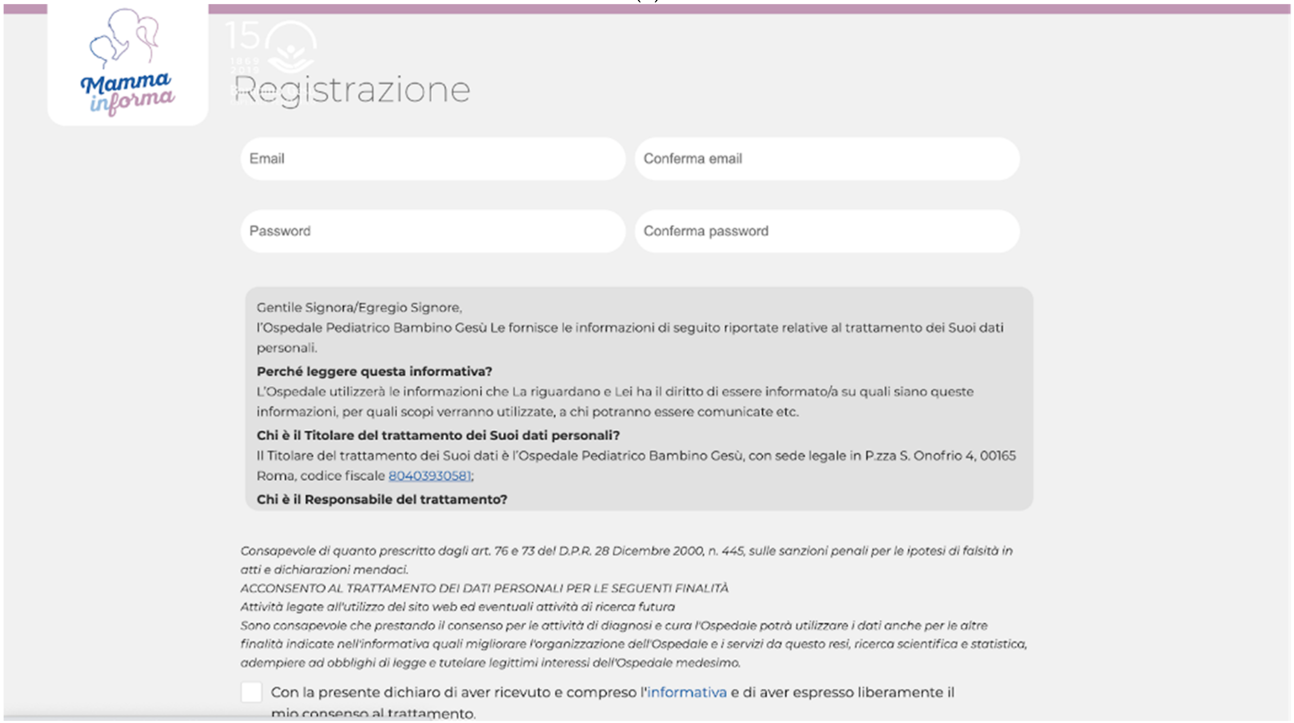
Mamma informa
Registrazione
Email
Conferma email
Password
Conferma password
Gentile Signora/Egregio Signore,
l'Ospedale Pediatrico Bambino Gesù Le fornisce le informazioni di seguito riportate relative al trattamento dei Suoi dati personali.
Perché leggere questa informativa?
L'Ospedale utilizzerà le informazioni che La riguardano e Lei ha il diritto di essere informato/a su quali siano queste informazioni, per quali scopi verranno utilizzate, a chi potranno essere comunicate etc.
Chi è il Titolare del trattamento dei Suoi dati personali?
Il Titolare del trattamento dei Suoi dati è l'Ospedale Pediatrico Bambino Gesù, con sede legale in P.zza S. Onofrio 4, 00165 Roma, codice fiscale 80403930581;
Chi è il Responsabile del trattamento?
Consapevole di quanto prescritto dagli art. 76 e 73 del D.P.R. 28 Dicembre 2000, n. 445, sulle sanzioni penali per le ipotesi di falsità in atti e dichiarazioni mendaci.
ACCONSENTO AL TRATTAMENTO DEI DATI PERSONALI PER LE SEGUENTI FINALITÀ
Attività legate all'utilizzo del sito web ed eventuali attività di ricerca futura
Sono consapevole che prestando il consenso per le attività di diagnosi e cura l'Ospedale potrà utilizzare i dati anche per le altre finalità indicate nell'informativa quali migliorare l'organizzazione dell'Ospedale e i servizi da questo resi, ricerca scientifica e statistica, adempiere ad obblighi di legge e tutelare legittimi interessi dell'Ospedale medesimo.
Con la presente dichiaro di aver ricevuto e compreso l'informativa e di aver espresso liberamente il mio consenso al trattamento.

(b)

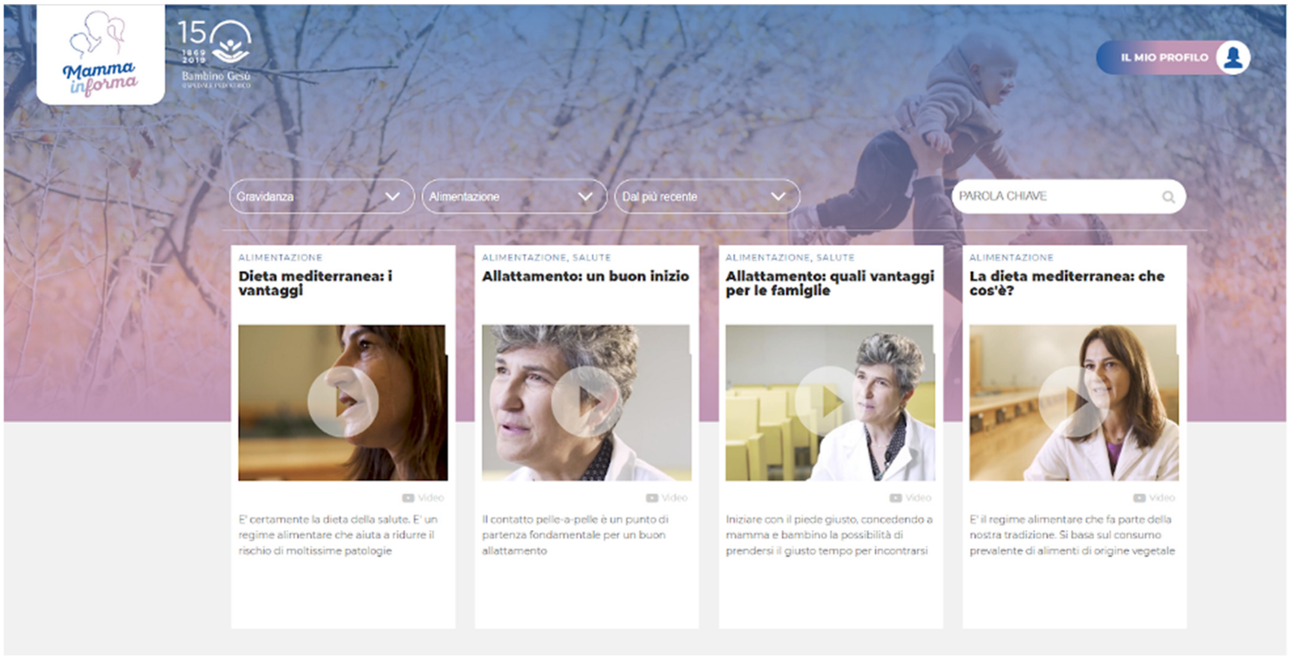

(c)

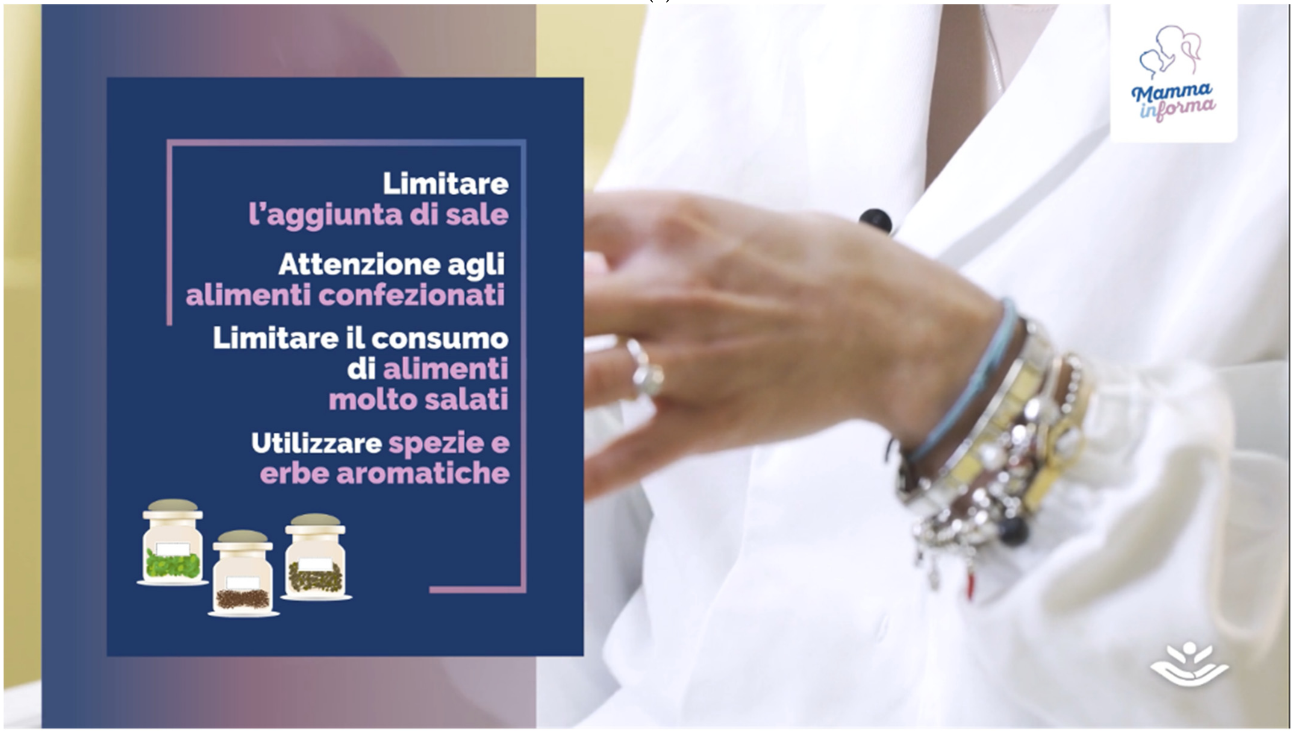

(d)

Figure S1. (**a**) Website's landing page; (**b**) Website's registration and consent form page; (**c**) Website's homepage; (**d**) Screenshot from one of the video interviews with graphics.

Table S3. Health Literacy Score's construction and percentage of answers for each question (n=1299).

	Score	n (%)
**How often is it difficult for you to understand written information about your health status (the content not the calligraphy)?**		
Always	1	15 (1.2)
Often	2	110 (8.5)
Sometimes	3	451 (34.7)
Rarely	4	478 (36.8)
Never	5	245 (18.9)
**How often do you need someone (a relative, a friend or someone else) helping you to read medical stuff (e.g. medical or clinical documentation, informed consent forms..)?**		
Always	1	82 (6.3)
Often	2	131 (10.1)
Sometimes	3	285 (21.9)
Rarely	4	343 (26.4)
Never	5	458 (35.3)
Rarely	4	478 (36.8)
**How confident are you filling out medical forms by yourself (e.g. declaration of not being pregnant before a radiography, detection of the presence of allergies before a vaccination..)?**		
Extremely	5	292 (22.5)
Quite a bit	4	450 (34.6)
Somewhat	3	366 (28.2)
A little bit	2	133 (10.2)
Not at all	1	58 (4.5)
**In general, how much is it easy or difficult for you to understand the results from medical statistical investigations?**		
Very easy	4	235 (18.1)
Easy	3	878 (67.6)
Difficult	2	181 (13.9)
Very difficult	1	5 (0.4)
**How much do you agree or disagree about this sentence? "In general, I rely upon numbers and statistics to make decisions about my health."**		
Strongly agree	4	99 (7.6)

Agree	3	726 (55.9)
Disagree	2	367 (28.3)
Strongly disagree	1	107 (8.2)
**Using information that your physician gives to you to make decisions about your pathology?**		
Very easy	4	248 (19.1)
Easy	3	932 (71.7)
Difficult	2	115 (8.9)
Very difficult	1	4 (0.3)

Values are expressed as number and percentage (n (%)) for categorical variables.

Table S4. Association between users' characteristics and videos' topic.

	Nutrition	Breastfeeding	Physical activity	Smoke, alcohol and recreational drugs	Pharmaceuticals	Child health	Paternal health
Age (Ref:<31)							
31-25	1.81 (0.57-2.46)	0.73 (0.53-1.52)	0.97 (0.42-2.25)	1.10 (0.53-2.29)	1.11 (0.41-2.97)	1.08 (0.52-2.26)	1.16 (0.60-2.23)
36-40	0.76 (0.35-1.62)	0.55 (0.25-1.19)	0.70 (0.29-1.69)	1.53 (0.73-3-21)	0.79 (0.28-2.24)	1.65 (0.77-3.55)	0.75 (0.38-1.50)
>40	1.23 (0.51-2.97)	0.55 (0.22-1.38)	0.62 (0.22-1.77)	1.45 (0.60-3.54)	0.94 (0.28-3.20)	**2.71 (1.09-6.70)**	1.11 (0.51-2.39)
Region (Ref:North)							
Center	0.67 (0.38-1.20)	0.66 (0.37-1.19)	0.56 (0.29-1.11)	1.05 (0.61-1.82)	0.75 (0.34-1.63)	1.18 (0.67-2.08)	1.38 (0.84-2.27)
South	0.93 (0.45-1.83)	**0.45 (0.21-0.95)**	0.51 (0.22-1.19)	0.85 (0.41-1.73)	0.62 (0.23-1.63)	1.03 (0.52-2.06)	0.98 (0.50-1.91)
Educational Level (Ref:Lower than Graduate School degree)							
Graduate School degree	1.10 (0.59-2.00)	1.21 0.65-2.27	1.93 (0.92-4.06)	1.01 (0.57-1.79)	0.57 (0.26-1.38)	1.21 (0.66-2.21)	1.15 (0.65-2.01)
Civil status (Ref:Unmarried)							
Married	0.58 (0.27-1.25)	0.91 (0.41-2.04)	0.50 (0.21-1.23)	0.69 (0.34-1.41)	1.82 (0.60-5.58)	1.35 (0.61-2.97)	0.58 (0.32-1.06)

Living together	0.65 0.28-1.51)	1.52 (0.63-3.64)	0.65 (0.25-1.72)	1.23 (0.59-2.58)	2.13 (0.63-7.19)	1.40 (0.59-3.31)	**0.46 (0.23-0.91)**
Divorced	0.72 (0.06-8.61)	6.53 (0.61-70.0)	0.77 (0.04-14.88)	1.67 (0.33-8.48)	11.43 (0.46-286.71)	0.84 (0.07-10.40)	0.60 (0.07-5.08)
Occupation (Ref: Full-time professional)							
Part-time professional	0.90 (0.45-1.80)	1.06 (0.52-2.18)	0.89 (0.39-2.00)	1.31 (0.71-2.44)	1.50 (0.58-3.85)	1.18 (0.59-2.35)	0.93 (0.47-1.85)
Unemployed	0.68 (0.33-1.41)	1.10 (0.53-2.27)	**0.37 (0.15-0.91)**	1.01 (0.50-2.05)	1.47 (0.55-3.92)	1.69 (0.84-3.40)	0,.90 (0.43-1.88)
Health professional (Ref:No)	1.07 (0.56-2.03)	0.79 (0.40-1.56)	0.89 (0.42-1.87)	0.77 (0.39-1.52)	**3.46 (1.40-8.54)**	0.75 (0.39-1.44)	1.20 (0.71-2.03)
HL (Ref:Low)							
Medium	0.90 (0.50-1.63)	1.43 (0.77-2.63)	0.87 (0.44-1.74)	1.20 (0.67-2.13)	1.41 (0.62-3.18)	**2.05 (1.12-3.75)**	1.53 (0.85-2.77)
High	1.20 (0.64-2.27)	1.60 (0.83-3.12)	1.00 (0.48-2.10)	1.04 (0.56-1.92)	1.30 (0.54-3.14)	1.59 (0.84-3.02)	1.60 (0.89-2.89)

Multilevel logistic regression model. The model is also adjusted for period. Values are expressed as Odd Ratio and 95% Confidence Interval (OR (CI 95%)). Statistical significance for $p<0.05$ (in bold).
